# Antigenic Site Immunodominance Redirection Following Repeat Variant Exposure

**DOI:** 10.3390/v14061293

**Published:** 2022-06-14

**Authors:** Lisa C. Lindesmith, Paul D. Brewer-Jensen, Michael L. Mallory, Mark R. Zweigart, Samantha R. May, Daniel Kelly, Rachel Williams, Sylvia Becker-Dreps, Filemón Bucardo, David J. Allen, Judith Breuer, Ralph S. Baric

**Affiliations:** 1 Department of Epidemiology, University of North Carolina at Chapel Hill, Chapel Hill, NC 27599, USA; pbj@email.unc.edu (P.D.B.-J.); mlmallor@email.unc.edu (M.L.M.); mrz@email.unc.edu (M.R.Z.); srmay@email.unc.edu (S.R.M.); sbd@email.unc.edu (S.B.-D.); 2 Department of Infection Biology, Faculty of Infectious and Tropical Diseases, London School of Hygiene and Tropical Medicine, London WC1E 7HT, UK; daniel.kelly@lshtm.ac.uk (D.K.); david.allen@lshtm.ac.uk (D.J.A.); 3 Department of Infection, Immunity and Inflammation, UCL Great Ormond Street Institute of Child Health, University College London, London WC1N 1EH, UK; rachel.williams@ucl.ac.uk (R.W.); j.breuer@ucl.ac.uk (J.B.); 4 Department of Genetics & Genomic Medicine, UCL Great Ormond Street Institute of Child Health, University College London, London WC1N 1EH, UK; 5 Department of Family Medicine, University of North Carolina at Chapel Hill, Chapel Hill, NC 27599, USA; 6 Department of Microbiology, National Autonomous University of Nicaragua-León (UNAN-León), León 21000, Nicaragua; fili_bucardo@hotmail.com; 7 Department of Microbiology, Great Ormond Street Hospital for Children NHS Foundation Trust, London WC1N 3JH, UK

**Keywords:** norovirus, neutralizing antibody, blockade antibody, immunodominance, variants of concern, antigenic seniority, immune imprinting, variant persistence

## Abstract

Human norovirus is a leading cause of acute gastroenteritis, driven by antigenic variants within the GII.4 genotype. Antibody responses to GII.4 vaccination in adults are shaped by immune memory. How children without extensive immune memory will respond to GII.4 vaccination has not been reported. Here, we characterized the GII.4 neutralizing antibody (nAb) landscape following natural infection using a surrogate assay and antigenic site chimera virus-like particles. We demonstrate that the nAb landscape changes with age and virus exposure. Among sites A, C, and G, nAbs from first infections are focused on sites A and C. As immunity develops with age/exposure, site A is supplemented with antibodies that bridge site A to sites C and G. Cross-site nAbs continue to develop into adulthood, accompanied by an increase in nAb to site G. Continued exposure to GII.4 2012 Sydney correlated with a shift to co-dominance of sites A and G. Furthermore, site G nAbs correlated with the broadening of nAb titer across antigenically divergent variants. These data describe fundamental steps in the development of immunity to GII.4 over a lifetime, and illustrate how the antigenicity of one pandemic variant could influence the pandemic potential of another variant through the redirection of immunodominant epitopes.

## 1. Introduction 

Human norovirus (HuNoV) is a leading cause of acute gastroenteritis, resulting in an estimated 200,000 deaths per year [1,2]. Although all age groups are susceptible to infection, children under five years of age are the most likely to require medical attention for symptoms of diarrhea, vomiting, and fever [3,4,5,6,7]. In lower- and middle-income countries (LMIC), an estimated 14–19% of diarrhea cases in children are associated with HuNoV [8]. Infection can be severe in developed nations as well, with an estimated 102 pediatric deaths occurring annually in the European Union [9]. This high burden of disease is similar to that of rotavirus before the implementation of vaccines [9], prompting the World Health Organization to prioritize the development of a HuNoV vaccine. To date, two vaccine candidates are in phase II clinical trials, although neither has yet reported on vaccine efficacy in children [10,11].

One of the leading obstacles to the development of a HuNoV vaccine is antigenic drift within the GII.4 genotype [12,13,14,15,16]. Despite there being more than 30 HuNoV genotypes currently observed [17], variants within the GII.4 genotype cause 50% to 70% of outbreaks, and caused pandemic waves of disease in the mid-1990s and again in 2002, 2004, 2006, 2009, and 2012 [18,19,20]. Each pandemic wave correlated with the replacement of the dominant GII.4 variant with a GII.4 variant exhibiting key changes in neutralizing antibody (nAb) epitopes, resulting in immune escape [12,13,14,15,16]. Propagating HuNoV in vitro is technically difficult and dependent upon primary human cells and virus-positive stool samples [21,22]. Thus, neutralizing antibody responses are commonly assessed with a surrogate neutralization assay that measures the ability of an antibody to block the interaction of HuNoV virus-like particles (VLPs) with a binding ligand. These “blockade” antibodies also neutralize the virus in vitro, and are a proposed correlate of protection and a key metric for vaccine studies [23,24,25,26,27,28,29,30].

**Figure 1:**
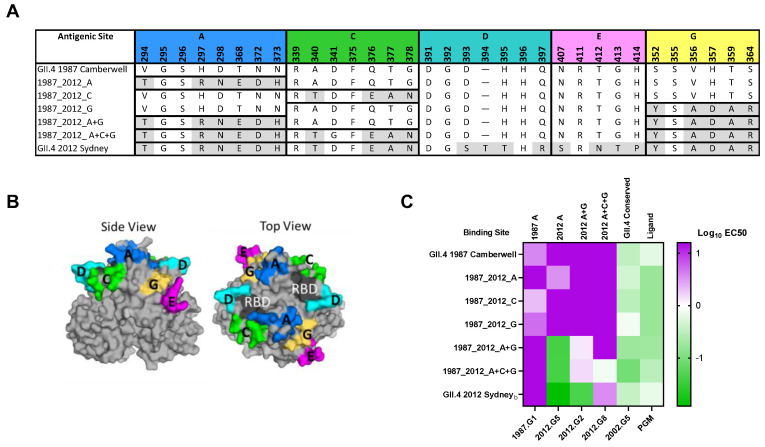
GII.4 2012 Sydney antigenic site chimera VLPs. (**A**) Residues of confirmed GII.4 hypervariable antigenic sites/epitopes in VLP studied here. Chimera VLPs are composed of GII.4 2012 Sydney antigenic sites in the backbone of GII.4 1987 Camberwell. (**B**) Antigenic sites in Panel A, color-coded on the structure of the GII.4 2012 Sydney dimer. Note: sites are colored as distinct entities, but antibodies may bind across sites. RBD: receptor binding domain. (**C**) VLPs characterized for epitope-specific nAb potency and ligand binding (Log EC50 µg/mL).

Bioinformatic analyses have predicted nine neutralizing antibody (nAb) antigenic sites, denoted A–I, on the GII.4 capsid protein [15,31]. Seven of these sites have been confirmed with monoclonal antibodies in the surrogate neutralization assay, and each is composed of multiple antibody epitopes, as defined by overlapping antibody binding footprints within and across antigenic sites [24,30,32]. Of these, sites A, C, D, E, and G are under positive selection, resulting in hypervariable regions in the virus capsid protein that define nAb antigenic sites (Figure 1) [15,33]. Antibodies to antigenic site/epitope A are dominant in sera from infected people, and site A is a primary target of isolated neutralizing monoclonal antibodies from humans and immunized mice [30,31,33,34]. Amino acid changes in site A highly correlate with new variant emergence. Recently, antigenic sites C and G were also reported to be highly correlated with new variant emergence, based on bioinformatic analysis [15,30]. Interestingly, sera and monoclonal antibodies from immunized animals indicate that antigenic site G may be co-dominant with antigenic site A after hyperimmunization with GII.4 2012 Sydney, leading to the hypothesis that a shift in immunodominance from site A and toward sites A and G or A+G may explain the unprecedented persistence of GII.4 2012 Sydney for the past 10 years [15,30]. A mechanism of how this shift in immunodominance would prevent the development of protective immunity to GII.4 2012 Sydney, allowing virus persistence, is unknown.

NAb responses in adults are driven by immune memory influenced by antigenic seniority [24,28]. Understanding the evolution of nAb responses in early childhood, when antigenic seniority is initiated, is key to understanding protection against emergent variants, and the rational design of vaccines. Here, in the first study to characterize the GII.4 nAb landscape in young children following natural norovirus infection, we define the relative impact of GII.4 antigenic sites A, C, and G on the serum nAb antibody response to GII.4 2012 Sydney in young children, and compare this response to time period-matched adult sera. We demonstrate that the serological antibody landscape is different between young children and adults, changes over time/virus exposure, and can be redirected and even broadened to cover divergent variants. These data describe fundamental steps in the development of immunity to GII.4 infection over a lifetime, and show how shifts in population immune focusing may mediate variant emergence.

## 2. Methods

Serum samples. Sera from first GII.4 infections were obtained from a birth cohort study in León, Nicaragua following children’s first RT-qPCR symptomatic GII.4 2012 Sydney infection. The children had a median age of 12 months (IQR 7–13 months) at infection, and the convalescent sera were all collected less than 7 months post-GII.4 infection. The study was approved by the Institutional Review Boards of the National Autonomous University of Nicaragua, León (UNAN-León, Acta Number 45, 2017) and the University of North Carolina at Chapel Hill (Study Number: 16-2079). Sera from children aged 1–2 and 15 years were obtained from Public Health England Seroepidemiology Unit (PHE SEU). Sera from adults collected in 2012–2014 (FluWatch) were obtained from the Health Survey for England Biobank. The use of coded serum samples was approved by the National Health Service Research Ethics Committee (reference 17/EE/0269), the London School of Hygiene and Tropical Medicine (reference LEO12196), and the University of North Carolina at Chapel Hill (18-0214). Sera collected from adults in 2019 were purchased from BioIVT (Hicksville, NY, USA). All sera were received coded, with no link back to donor identification, and were heat-inactivated for 30 minutes at 56 °C before use.

Structural homology modelling. Blockade/neutralizing antibody antigenic sites were mapped onto the surface of a GII.4 2012 Sydney P domain dimer (PBD 4WZT) and the residues color-coded with the PyMOL Molecular Graphics System, Version 2.0 Schrödinger, LLC (New York, NY, USA). 

Virus-like particles (VLPs) production. ORF2 genes corresponding to the capsid protein sequence of GII.4 2012 Sydney (AGJ52172.1) and GII.4 1987 Camberwell (AAK50355.1), GII.4 1997 Grimsby (AFJ04707.1), GII.4 2002 Farmington Hills (AFJ04708.1), GII.4 2006 Den Haag (AFU92665), GII.4 2009 New Orleans (ADQ43781.1), GII.4 2019 Hong Kong (QEL43936.1), and chimeric VLPs were synthesized by Bio Basic Inc (Markham, ON) and inserted directly into the Venezuelan equine encephalitis virus replicon vector for the production of VLPs in baby hamster kidney cells (ATCC CCL-10, Manassas, VA, USA) as described [35,36]. GII.4 2012 Sydney_b_ (MZ376651) VLPs were produced from baculovirus vectors as described [37]. These additional VLPs were included for comparison between strains of the same variant in the epitope correlation analyses. The particle integrity of the VLPs was verified by ligand and antibody binding, and visualization of ~40 nm particles by electron microscopy. 

Antibody blockade of VLP–ligand binding assays. The surrogate neutralization assay, based on measuring the ability of antibody to block VLP-to-ligand binding, was performed as described previously [38,39]. First, 0.25 µg/mL VLPs were pre-treated with decreasing concentrations of sera for 1 h, then transferred to pig gastric mucin (PGM, 10 µg/mL, Sigma Aldrich, St. Louis, MO, USA)-coated plates for 1 h, and bound VLPs were detected with rabbit anti-VLP sera and visualized with anti-rabbit-IgG-HRP (Sigma Aldrich, St. Louis, MO, USA). The percentage of control binding was compared to no serum pre-treatment. Mean ID_50_ (inhibitory dilution, 50%) titers and 95% confidence intervals (95% CI) were determined from log(inhibitor) verses normalized response-variable slope curve fit in GraphPad Prism 9.1.2 (La Jolla, California, USA) [28,40]. Sera that did not block at least 50% of VLP binding to ligand at the lowest dilution tested (40) were assigned a titer of 20 for statistical analysis. 

Statistical analysis. Blockade antibody titer comparisons and Spearman correlation analyses were performed using GraphPad Prism 9.1.2 [28,33]. ID_50_ were Log transformed for all analyses. Wilcoxon matched-pairs signed-rank test was used when comparing between VLPs for a serum set. A difference was considered significant if *p* < 0.05.

## 3. Results

**Figure 2:**
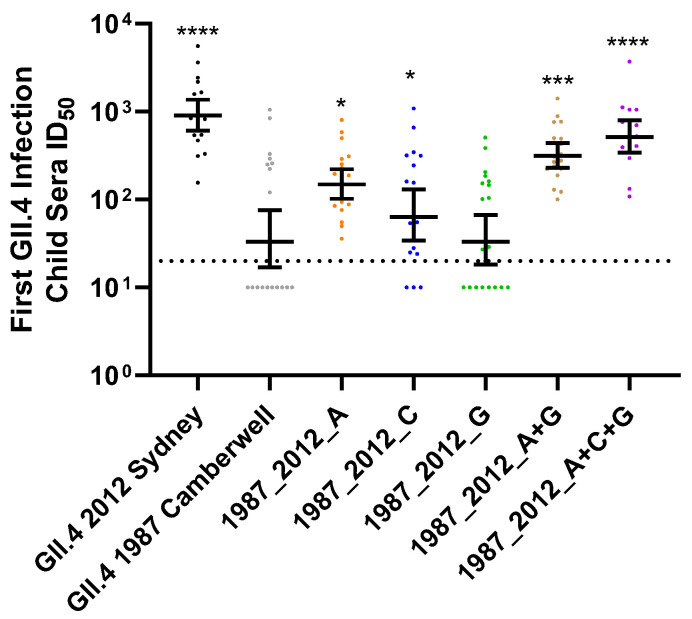
GII.4 Sydney infection in young children induces nAb responses to multiple overlapping antigenic sites. Sera from children (*n* = 20) with an RT-qPCR confirmed first symptomatic GII.4 infection, typed as GII.4 2012 Sydney, were evaluated for nAb titer to GII.4 2012 Sydney, GII.4 1987 Camberwell, and antigenic site chimera VLPs comprised of GII.4 2012 Sydney epitopes in the backbone of GII.4 1987. Y-axis: reciprocal of the serum dilution at 50% inhibitory dose. Marker: individual child. Line: geometric mean titer. Error bars: 95% confidence intervals. Dashed line: limit of detection. * *p* = 0.02, *** *p* = 0.0001, **** *p* < 0.0001 compared to GII.4 1987 Camberwell.

Development of immunity to GII.4 HuNoV is complex, complicated by pervasive exposure to antigenically diverse viruses, and by immune imprinting that favors memory responses to previous infecting variants over novel responses to a current infecting variant in adults [24,28]. While these concepts have been described at the antigenic site level in adults, nothing is known about the development of antigenic site-specific immunity in children. Focusing on antigenic sites with high correlation to new variant emergence [15], we developed a panel of virus-like particles (VLP) comprised of GII.4 2012 Sydney antigenic site A, C, or G, exchanged singly or in combination, into the backbone of a divergent GII.4 variant detected in 1987 (Camberwell), creating epitope-chimera VLPs (Figure 1). Chimera VLPs retained ligand binding ability and sensitivity to epitope-specific monoclonal antibody (mAb) neutralization, transferred according to known antibody patterns using the surrogate neutralization assay based on the antibody blockade of ligand binding (Figure 1C) [24,41,42,43]. Most mAbs depended on residues within site A for neutralization, but inclusion of residues from additional sites improved nAb potency. We then compared the nAb response to each site with sera collected from young children enrolled in a birth cohort study, sampling symptomatic and asymptomatic acute gastroenteritis infection [44]. Sera were collected every six months during the first thirty six months of life. In all cases, GII.4 2012 Sydney was confirmed as the infecting GII.4 variant. As expected, the cohort mounted robust GII.4 Sydney nAb (GMT 948), with limited cross-reactivity to the ancestral GII.4 1987 Camberwell (GMT 47) (Figure 2, Table S1). Compared to GII.4 1987 Camberwell, nAb potency was gained by genetic replacement of residues comprising individual antigenic sites A (GMT 158) and C (GMT 79), but not G (GMT 43). Exchange of A (but not C or G) alone or in combination resulted in significant increases in nAb responses to GII.4 1987 Camberwell. nAb potency improved with combinatorial reconstitution of antigenic sites A+G (GMT 328), with maximum titer gained for GII.4 1987 Camberwell displaying GII.4 Sydney sites A+C+G (GMT 540). These data indicate that, following the first GII.4 2012 Sydney infection in young children, antigenic site A is the most dominant site among single antigenic sites tested, and antibodies that cross antigenic sites are common and potent nAbs. 

**Figure 3:**
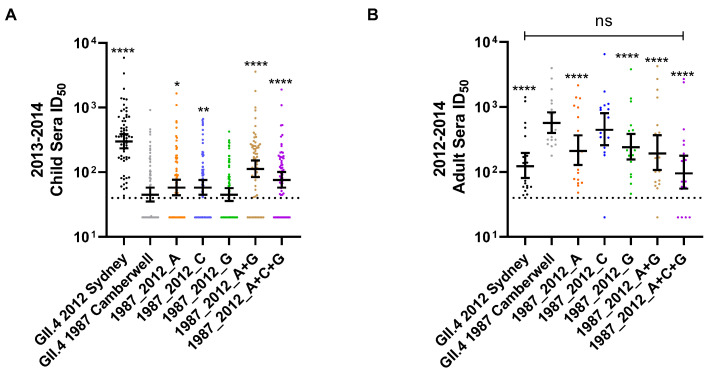
Antigenic site-specific nAb responses vary by age/exposure history. Surveillance sera from children ages ≤ 5 years (*n* = 65) (**A**) and adults ≥ 15 years (*n* = 21) (**B**) were evaluated for nAb antibody titer to GII.4 2012 Sydney, GII.4 1987 Camberwell, and antigenic site-chimera VLP comprised of GII.4 2012 Sydney antigenic sites in the backbone of GII.4 1987 Camberwell. Only samples with titer ≥ 50 to either GII.4 2012 Sydney or 1987 Camberwell were included in analyses to exclude subjects who are genetically resistant to GII.4 infection. Y-axis: reciprocal of the serum dilution at 50% inhibitory dose. Marker: one individual. Line: geometric mean titer. Error bars: 95% confidence intervals. Dashed line: limit of detection. * *p* = 0.02, ** *p* = 0.004, **** *p* < 0.0001 compared to GII.4 1987 Camberwell.

Immunologically naïve, genetically susceptible [45] young children are likely vulnerable to infection from any fit GII.4 variant. The pandemic potential of a variant is dependent upon its immune evasion within a population with pre-existing GII.4 immunity, i.e., older children and adults. To investigate the antigenic site-specific response following GII.4 2012 Sydney infection in GII.4-exposed cohorts, we next evaluated sera collected from a surveillance study conducted at the peak of Sydney circulation in the United Kingdom in 2012–2014 [46]. To exclude individuals who are genetically resistant to GII.4 infection, we only included individuals with ID50 titer ≥ 50 to either GII.4 2012 Sydney or GII.4 1987 Camberwell [47]. For further analyses, we combined the children of five years and younger into one group, as children ≤ 5 years bear the largest burden of norovirus infection [18]. As shown with sera from young children collected post-GII.4 2012 Sydney infection (Figure 2), surveillance sera from young children had high GII.4 2012 Sydney titer in 2013–2014 (GMT 327), with relatively low titer to GII.4 1987 Camberwell (GMT 51) (Figure 3A, Table S2). Exchange of antigenic sites A (GMT 67) or C (GMT 66), but not G (GMT 50), increased sera nAb potency for GII.4 1987 Camberwell. Any VLP that included site A had higher nAb titer, with the peak titer to 1987_2012_A+G (GMT 129), supporting the dominant role of antibodies that bind to A and span across to site G in children with pre-existing GII.4 exposure, including GII.4 2012 Sydney, as described for first GII.4 infections. 

While GII.4 2012 Sydney antigenic site-specific responses in convalescent sera from young children post-first infection support the dominance of nAb for site A, surveillance sera from the adult cohort identified a broadening of immunity across antigenic sites A and G or A+G (Figure 3B, Table S3). The nAb of adult sera to GII.4 variants is heavily impacted by immune imprinting, specifically antigenic seniority, characterized by preferential back-boosting of nAbs to ancestral variants [24,28]. Accordingly, adult sera have higher nAb titer to ancestral GII.4 1987 Camberwell (GMT 597) than to GII.4 2012 Sydney (GMT 136); therefore, a reduction in ID50, compared to GII.4 1987 Camberwell, defines a targeted antigen site. For adult sera, exchange of site A (GMT 238) or site G (GMT 262) singly or in combination resulted in loss of nAbs compared to GII.4 1987 Camberwell (Figure 3B). The influence of antigenic sites A and G were not different from each other, and greater than C (GMT 496). Change in single site C was not significant from GII.4 1987 Camberwell, but A+C+G (GMT 114) had a greater impact than A+G (GMT 227), indicating site C nAbs are present but subdominant. nAb titer between GII.4 1987_2012_A+C+G (GMT 114) and GII.4 2012 Sydney (GMT 136) was not different in adults.

**Figure 4:**
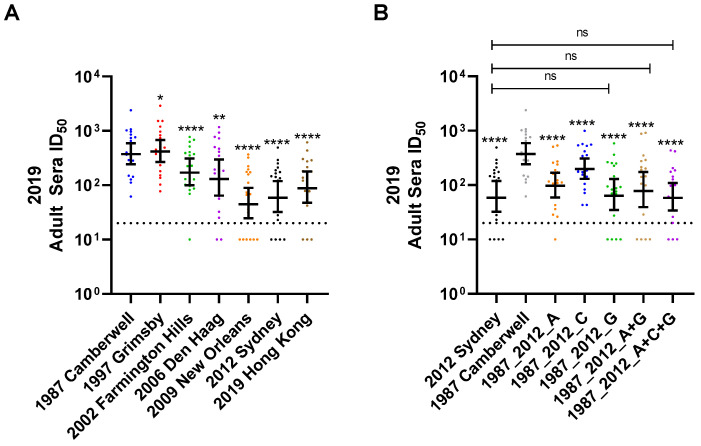
Variant and antigenic site-specific nAb responses in contemporary adult sera. Sera from adults collected in 2019 (*n* = 20) were evaluated for nAb titer to a panel of GII.4 variants (**A**) or antigenic site chimera VLPs comprised of GII.4 2012 Sydney antigenic sites in the backbone of GII.4 1987 Camberwell (**B**). Only samples with titer ≥ 50 to either GII.4 2012 Sydney or 1987 Camberwell were included in the analysis to exclude subjects who are genetically resistant to GII.4 infection. Y-axis: reciprocal of the serum dilution at 50% inhibitory dose. Marker: individual adult. Line: geometric mean titer. Error bars: 95% confidence intervals. Dashed line: limit of detection. * *p* = 0.02, ** *p* = 0.001, **** *p* < 0.0001 compared to GII.4 1987 Camberwell.

These data establish that antigenic sites A and G or A+G comprise the key nAb sites on GII.4 variants in adult sera collected in 2012–2014, during the peak of GII.4 2012 Sydney circulation, and possibly support the hypothesis that GII.4 2012 Sydney exposure is a driver of development of nAbs directed to antigenic site G in adults with pre-exposure history, pinpointing a potential mechanism of immune evasion of GII.4 Sydney, if antigenic site G antibodies are insufficient to provide protection. The limited volume of the archival sera limited additional analyses of the epitope-specific response in these cohorts; therefore, we evaluated a set of commercial sera collected from adults in 2019 to further investigate antigenic site-targeting in adults. The nAb titer to ancestral variants was higher than the nAb response to contemporary variants (Figure 4A, Table S4). Notably, even after seven years of GII.4 2012 Sydney circulation, adult sera titer to Sydney was relatively low (GMT 72) compared to ancestral variants (GMT 401 for GII.4 1987 Camberwell), indicating that the low titer observed in 2012–2014 adult sera was not reflective of a lack of GII.4 Sydney exposure, but instead a result of decreased responses, evidence of antigenic seniority. In agreement with the 2012–2014 adult sera, exchange of single antigenic sites A (GMT 110) or C (GMT 212) or G (GMT 79) impacted nAb potency. Antigenic sites A and G were not different from each other in terms of influence, and the influence of each was greater than C. Neutralizing Ab potency did not differ between chimera VLP that included sites A and G (GMT 100) or A, C, and G (GMT 69) and GII.4 2012 Sydney. While single site A (GMT 110) trended toward GII.4 2012 Sydney titer, single site G (GMT 79) was not different from GII.4 2012 Sydney titer (GMT 72). These data support the broadening of nAb epitope recognition following GII.4 Sydney infection to include site G and/or antibodies that reach across sites A and G, and suggest repeated GII.4 2012 Sydney exposure may drive more nAbs toward sites G or A+G (Figure 4B). 

**Figure 5:**
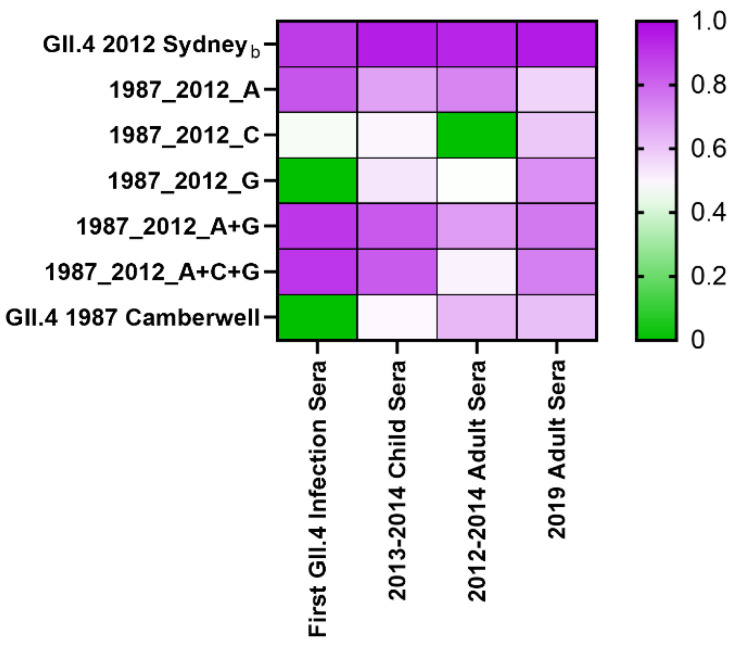
Repeat GII.4 Sydney exposure correlates with antigenic site G nAb titer. nAb titers to GII.4 2012 Sydney and antigenic site-specific responses were compared by Spearman correlational analysis, and the r value was determined. Pairs with insignificant correlation were assigned r = 0. A related GII.4 Sydney strain (GII.4 2012 Sydney_b_) was included in the analyses for comparison.

Correlational analyses of antigenic site-specific nAb titers between cohorts support the hypothesis that GII.4 2012 Sydney repeat exposure is associated with the development of nAbs at GII.4 2012 Sydney antigenic site G (Figure 5). Post-first GII.4 infection in children, nAb responses were driven by epitope A (r = 0.84, *p* < 0.0001) as a single site or in any combination (r = 0.90, *p* < 0.0001). In the cohorts with multiple GII.4 exposures, as the correlation with site A decreased, the correlation with site G increased. In surveillance sera from children in 2013–2014, GII.4 2012 Sydney titer moderately correlated with site A (r = 0.67, *p* < 0.0001) and site G (r = 0.53, *p* < 0.0001). Higher correlation for site A (r = 0.74, *p* < 0.001) compared to site G (r = 0.48, *p* = 0.03) was observed for adult sera from 2012–2014. In contrast, adult sera from 2019 had the highest correlation between GII.4 2012 Sydney and antigenic site G (Spearman r = 0.71, *p* = 0.0004) compared to site A (r = 0.57, *p* = 0.009). Antigenic site correlation was poor to moderate across the cohorts for site C (r = 0.46–0.60, *p* < 0.006). Antigenic sites A+C+G in combination were not more highly correlated with GII.4 2012 Sydney titer than the combination of A+G in any cohort, indicating that antigenic site C is subdominant. These data indicate that antigenic sites A and G shift in relative immunodominance after repeated GII.4 2012 Sydney exposure, but antigenic site C does not. 

**Figure 6:**
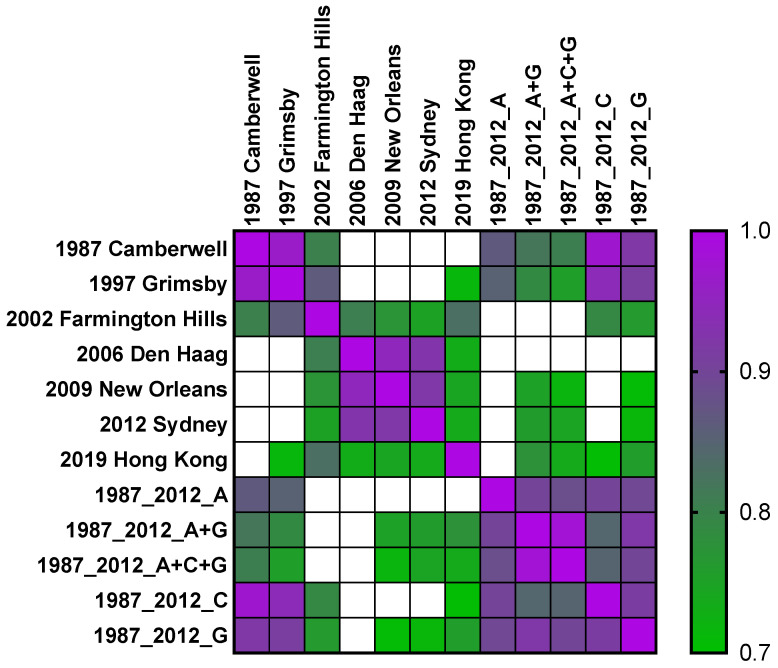
Neutralizing antibody to GII.4 2012 Sydney antigenic site G correlates with nAb to antigenically distinct GII.4 variants. nAb titers to GII.4 variants and antigenic site chimera VLPs were compared by Spearman correlational analysis, and the r value was determined. All pairings were significant. Pairs with high correlation (r ≥ 0.7) are shown.

To determine if the redirection of nAb from site A to both sites A and G corresponded with the breadth of GII.4 nAb measured in the 2019 adult sera, we compared the titer of each antigenic site with the titer to each GII.4 variant. As expected for variants, each GII.4 significantly correlated with the others, with two groups of high correlation (r ≥ 0.7, *p* ≤ 0.005) (Figure 6): ancestral variants (1987 Camberwell, 1997 Grimsby, and 2002 Farmington Hills) and contemporary variants (2006 Den Haag, 2009 New Orleans, and 2012 Sydney) [43,48]. Among the single antigenic sites, A was the least likely to highly correlate across variants. Exchange of site A decreased or ablated the high correlation between ancestral variants without achieving high correlation to contemporary variants. Conversely, exchange of site G resulted in a high correlation with all the variants tested, except GII.4 2006 Den Haag, which was slightly lower (r = 0.67, *p* = 0.001). Notably, GII.4 2019 Hong Kong, a divergent variant that has only recently been detected and is not yet widely circulating in adults [49,50], also highly correlated with other GII.4 variants, with the best correlation to GII.4 2002 Farmington Hills (r = 0.83, *p* < 0.0001), not the contemporary variants. GII.4 2019 Hong Kong did not highly correlate with GII.4 1987 Camberwell (r = 0.69, *p* = 0.001) or GII.4 1987_2012_A (r = 0.63, *p* = 0.003). GII.4 2019 Hong Kong did highly correlate with Site G (r = 0.76, *p* < 0.0001) and any combination that included site G. These data, across multiple GII.4 pandemic and endemic variants, indicate an association between the development of nAbs to antigenic site G and the breadth of GII.4 nAb potency. Immunodominance redirection from antigenic site A to sites A and G or A+G by GII.4 2012 Sydney virus exposure, and the subsequent broadening of GII.4 nAb responses across divergent variants, may explain the immunological mechanism for the absence of emergence of a novel GII.4 variant in adults, extending the period of GII.4 Sydney 2012 persistence. 

## 4. Discussion

HuNoV infections peak between the ages of 6 and 18 months and are characterized by repeat infection with diverse genotypes [51]. However, repeat infection with the same genotype or the same GII.4 variant is rare, indicating that protective immunity does control disease but may be genotype/variant specific, particularly in the young [52]. Previous studies in adults have demonstrated that GII.4 nAb responses are biased toward recall of memory B cells primed from previously circulating GlI.4 variants [24,28]. Recalled nAbs predominantly recognize antigenic sites A or A+G, as measured by a surrogate neutralization assay [13,24]. The potency, durability, breadth, or epitopes of nAbs have not been characterized in children who lack the extensive pre-exposure history of adults. Understanding how immunity develops in response to first and subsequent GII.4 exposures may be key to the development of broadly protective HuNoV pediatric vaccines. Here, leveraging a unique set of time-ordered child and adult sera cohorts, we illustrate key differences between children and adults in nAb responses to GII.4 variants.

In young children, nAb responses skew toward sites A and C of GII.4 2012 Sydney, with little response to site G, although some individual children did respond to G. In this cohort, combining sites C and/or G with site A improved nAb titer but did not recapitulate the titer to GII.4 2012 Sydney, indicating that antibodies to additional antigenic sites are also constituents of the nAb repertoire of young children. In contrast, in adult sera from the peak of GII.4 2012 Sydney circulation (2012–2013 adult cohort), antigenic sites A and G co-dominate. Combining sites A, C, and G reconstitutes GII.4 2012 Sydney titer in this cohort, indicating that the nAb response focuses onto a more limited number of key sites with repeated exposure to different GII.4 variants over a lifetime. In all cohorts studied, nAbs that span multiple sites are major contributors to the serum nAb response. 

The focusing of nAb on sites A, G, and A+G was confirmed in adult sera from 2019. Notably, the relative dominance of nAbs to site A verses site G changed in the 2019 adult sera, compared to the nAb repertoire in the earlier adult cohort, resulting in a co-dominance between A and G. This shift likely correlates with repeat exposure to GII.4 2012 Sydney, although it may represent Sydney recall of GII.4 1997 Grimsby nAb, as has been described post-GII.4 vaccination [24]. Adults in 2012–2014 would have been exposed to many GII.4 variants, and site G played a minor role in the nAb response in this earlier cohort compared to site A. Meanwhile, GII.4 2012 Sydney was the only dominant GII.4 variant between 2012 and 2019. Hyperimmunization of animals with GII.4 2012 Sydney supports the idea that this variant is the driver of site G, as well as sites A and A+G nAb, although a similar response to other variants cannot be ruled out [30]. 

Immune focusing on select epitopes and nAbs that cross antigenic sites are not unusual [24,53,54,55]. The most surprising finding of this study is the correlation between nAbs to site G and the breadth of GII.4 nAb potency. Similarly, epitope dominance redirection for the B.1.351 SARS-CoV-2 variant has been proposed to result in nAbs that are less sensitive to mutations [56]. Neutralizing Ab to site G correlated with nAb across a wide spectrum of antigenic variants, which have circulated widely as pandemic viruses and rarely as endemic viruses. Further studies of site G are warranted to define potential therapeutic cross-neutralizing monoclonal antibodies and to inform vaccine design. A hypervariable site conferring cross-nAbs has been demonstrated previously. Vaccination with a heterologous GII.4 boosted nAbs to site A in both ancestral and contemporary GII.4 variants [24,28]. Although site A is hypervariable, cross-variant nAbs to site A have been mapped to conserved residues within the site and structurally conserved residues between variants [24,31]. Regardless of the mechanism of nAb breadth, the consequence of nAbs to site G appears to be suppressing the emergence of a new dominant GII.4 variant capable of replacing GII.4 2012 Sydney, based on escape from population immunity. 

This hypothesis may explain why a new GII.4 variant has not emerged since 2012, but it does not explain why GII.4 2012 Sydney persists in adult populations. One would assume nAbs to site G would be protective and, as population immunity reached saturation, the variant would eventually become extinct, as occurred with previous pandemic GII.4 variants with site A immunodominance. How could site G nAbs be protective against other variants and not GII.4 2012 Sydney? Although speculative, pre-existing nAbs to site A may be driving the lack of protective immunity to GII.4 2012 Sydney, as demonstrated for repeat influenza vaccination [57]. Monoclonal antibodies to site A of the closely related variant GII.4 2006 Den Haag bind to GII.4 2012 Sydney, but are less potent at neutralizing GII.4 2012 Sydney [16]. Following GII.4 2012 Sydney infection, high affinity memory B cells to site A of GII.4 2006 Den Haag may be preferentially selected for expansion instead of naive B cells specific for GII.4 2012 Sydney, compromising GII.4 2012 Sydney protective immunity and favoring virus persistence. Repeat exposure to GII.4 2012 Sydney could allow for the expansion of B cells to other less dominant sites, including site G. However, if site A is bound by GII.4 2006 Den Haag Abs, which are unable to neutralize the GII.4 2012 Sydney virus, but able to block binding of GII.4 2012 Sydney-specific nAbs, GII.4 2012 Sydney immunity will continue to be ineffective, and the virus will continue to circulate. As children born since 2012 age, the population serological repertoire to GII.4 will transition to one primed against GII.4 2012 Sydney, and viruses of this variant will become extinct, as variant-specific immunity is likely protective. 

HuNoV immunity is complex, confounded by antigenic variants and immune imprinting, features driven by both the virus and the host. These complexities translate into functional differences in nAb responses between young children and adults, indicating the potential pitfalls of using adults to study correlates of protection or vaccine outcomes for a primarily pediatric illness. Our findings highlight an overwhelming need to study pediatric cohorts to understand HuNoV immunity. Birth cohorts followed long-term (>10 years), coupled with serological repertoire profiling over time, would allow for the tracking of the development of immunity by age and exposure, clarifying how GII.4 exposures impact nAb responses to subsequent GII.4 exposures (infection or vaccination).

The observation that young children likely recognize a more diverse set of nAb epitopes compared to adults may provide optimism for developing a vaccine capable of providing cross-GII.4 immunity. Here, limited sample volume limited our ability to fully map the nAb landscape in any of the cohorts. Additional nAb antigenic sites/epitopes are known or proposed and should be evaluated, especially in children, a significant hurdle based on limitations of sample collection. However, further study of nAb responses in young children may put us in a position to apply rational vaccine design approaches to HuNoV with the end goal of relieving children from the burden of HuNoV disease. 

## Data Availability

All study data are reported within the manuscript and supplemental materials.

## Supplementary Materials

The following supporting information can be downloaded at: https://www.mdpi.com/article/10.3390/v14061293/s1, Table S1. Antigenic site-specific ID50 in children post-first GII.4 infection. Table S2. Antigenic site-specific ID50 in Child Sera 2013–2014. Table S3. Antigenic site-specific ID50 in Adult Sera 2012–2014. Table S4. Variant and antigenic site-specific ID50 for 2019 adult sera.
